# Effect of Calcium Channel Blockers on Lower Urinary Tract Symptoms: A Systematic Review

**DOI:** 10.1155/2017/4269875

**Published:** 2017-10-16

**Authors:** Muhammad Salman, Amer Hayat Khan, Syed Azhar Syed Sulaiman, Junaid Habib Khan, Khalid Hussain, Naureen Shehzadi

**Affiliations:** ^1^Discipline of Clinical Pharmacy, School of Pharmaceutical Sciences, Universiti Sains Malaysia, Penang, Malaysia; ^2^Punjab University College of Pharmacy, University of the Punjab, Lahore 54000, Pakistan; ^3^Department of Urology (Unit-I), Mayo Hospital, Lahore, Pakistan

## Abstract

**Background:**

Numerous medications are known to be associated with the development of lower urinary tract symptoms (LUTS). One such medication group is calcium channel blockers (CCB).

**Objective:**

To critically examine the literature regarding the involvement of CCB in manifestation of LUTS in humans.

**Methods:**

A systematic literature search was conducted on PubMed, SciELO, Scopus, and OpenGrey databases to find all potentially relevant research studies before August 2016.

**Results:**

Five studies met the inclusion criteria and were included in this review. Three out of five studies stated that CCB were involved in either precipitation or exacerbation of LUTS. As for the remaining two studies, one study found out that only the monotherapy of CCB was associated with increased prevalence of nocturia and voiding symptoms in young females, whereas the other study reported an inverse association of CCB with LUTS. The methodological quality of studies was considered high for four studies and low for one study.

**Conclusion:**

Healthcare providers should make efforts for an earlier identification of the individuals at risk of LUTS prior to the commencement of CCB therapy. Moreover, patients should be counselled to notify their healthcare provider if they notice urinary symptoms after the initiation of CCB.

## 1. Introduction

Lower urinary tract symptoms (LUTS) is an umbrella term that was first coined in 1994 to disassociate urinary symptoms in male from any implied specific site of origin of the symptoms, such as prostate [1]. LUTS encompasses all urinary symptoms, namely, voiding, storage, and postvoiding [2], and this term corroborates well with the earlier classification proposed by Wein [3, 4] who suggested that urinary disorders would be more elegantly characterized as “failure to store” or “failure to empty.” These symptoms are common and troublesome and have a negative impact on patients' quality of life (QOL). LUTS are considered to be progressive, age-related, non-gender-specific, non-organ-specific group of symptoms [2] and have been associated with various diseases, surgeries, and medications. One such medication group is calcium channel blockers (CCB) as the blockade of L-type calcium channels in detrusor muscles not only inhibits the bladder contraction [5–7], but also increases the duration to reach maximal bladder pressure and reduces maximal power of contraction, maximal rate of emptying, and rate of bladder filling which may lead to polyuria, micturition frequency, micturition disorder, and nocturia [8].

The current review was conducted to answer the question “Is there a significant relationship between the use of CCB and manifestation of LUTS in humans?” This review may help the healthcare providers in appropriate selection of CCB having minimal or no urological effects.

## 2. Methodology

### 2.1. Administrative Information

For the current systematic review, we followed the protocols and checklist of Providing Innovative Service Models and Assessment (PRISMA) [9] and PRISMA-Protocol 2015 [10, 11]. We were not able to conduct meta-analysis due to wide diversity in the studies reporting association of CCB with LUTS. Therefore, the articles were analyzed descriptively.

### 2.2. Study Selection

A systematic literature search was conducted by two investigators (MS and NS) on PubMed, Scopus, Scientific Electronic Library Online (SciELO), and OpenGrey databases to find all potentially relevant publications before August 2016. The following categories of words/terms and their synonyms were used: calcium channel blockers, antihypertensive drugs, lower urinary tract synonyms, and urination disorders. The reference lists of screened publications were also checked to identify further relevant studies. Moreover, if required, corresponding authors of the included studies were also contacted through email.

### 2.3. Inclusion and Exclusion Criteria

All the original research articles that evaluated the association between calcium channel blockers and lower urinary tract symptoms in adults (>18 years) were included in this review. Literature reviews, editorials, commentaries, case reports, and conference abstracts were excluded, as were the studies with patients on medications other than calcium channel blockers and patients <18 years of age and studies published in a language other than English.

### 2.4. Data Extraction

After eliminating duplicates, two investigators (MS and NS) reviewed each article independently. Discrepancies were discussed and agreement was achieved by consensus and opinion of a third investigator (AHK) was requested where necessary. The full-text of all articles which met the inclusion criteria was obtained. Relevant data were extracted and tabulated.

### 2.5. Quality Assessment

In the current review, the quality of included studies was assessed independently by two investigators (MS and KH) using Newcastle-Ottawa scale (NOS) for cohort studies [12] and a modified version for cross-sectional studies developed by Herzog et al. [13]. Newcastle-Ottawa scale consists of 3 parameters of quality: selection (4 points for cohort studies and 5 points for cross-sectional studies), comparability (2 points), and outcome assessment (3 points). Studies with scores of ≥7 were considered as high quality studies and of 5-6 as moderate quality [14].

### 2.6. Case Definition

In the current review, LUTS encompass all voiding (weak stream, splitting or spraying, intermittency, hesitancy, straining, and terminal dribble), storage (increased daytime urinary frequency, nocturia, urgency to urinate, and urinary incontinence), and postmicturition symptoms (sensation of incomplete bladder emptying and postmicturition dribbling) [2].

## 3. Results

### 3.1. Search Results

**Figure 1:**
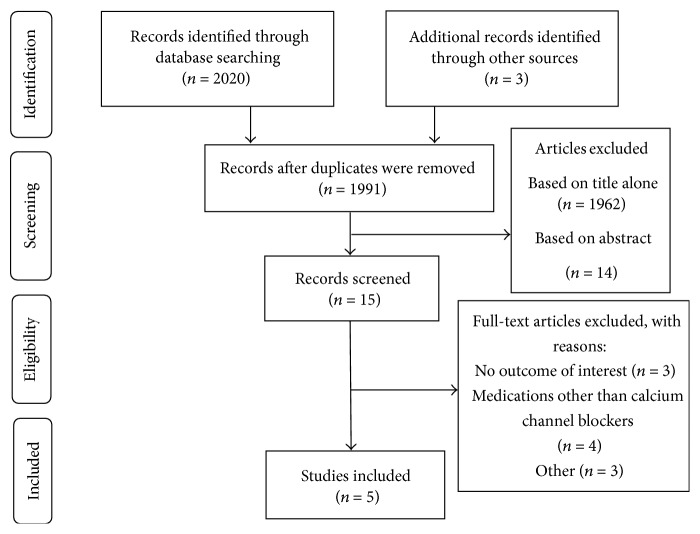
Flow diagram of the identification of the studies for inclusion in the systematic review.

As depicted in Figure 1, we identified two thousand and twenty-three studies. After removing the duplicates and after exclusion of studies based on the examination of the titles and abstracts, fifteen studies were selected for the review of the full-text. After the detailed evaluation of fifteen articles, 5 studies were included in the final review. Among the 10 excluded studies, 3 had no outcome of interest, 4 evaluated the association of urinary symptoms with other medications, one study reported the prevalence of commonly prescribed medications potentially contributing to urinary symptoms among geriatrics seeking care for incontinence, one study was published from Elhebir dissertation [16], and one dissertation from which the manuscripts of Hughes et al. was published [15].

### 3.2. Study Characteristics

Characteristics of the included studies are shown in Table 1. All the studies were published in the last eight years (oldest in 2009 and latest in 2013). Of the five included studies, two were conducted in Australia [15, 16] whereas the rest of the studies were conducted in the USA [17], Japan [18], and Netherlands [19]. The number of study population ranged from 38 to 3790, with majority of the studies (3/5) involving only males [15, 18, 19]. Three out of five studies were undertaken on individuals aged >40 years [15, 16, 19], whereas one study [17] included individuals ranging from 30 to 79 years of age and one study did not specify the criterion of age [18]. International prostate symptom score was the predominantly used research tool to assess the frequency and severity of LUTS [15, 16, 18, 19]. Only one study used the American Urological Association-Symptom Index for the evaluation of LUTS [17].

### 3.3. Association of Calcium Channel Blockers with Lower Urinary Tract Symptoms

The relationship of CCB with LUTS is shown in Table 2. The number of CCB-users varied between the studies [minimum 38 and maximum 207 (54 on CCB monotherapy and 153 on CCB and other antihypertensive drugs)]. Of all the included studies, two studies [15, 16] reported the types of CCB that were evaluated for their association with LUTS. Three out of five studies [15, 16, 18] reported a significant association of CCB use with LUTS whereas one study [17] reported that only monotherapy of CCB was associated with increased prevalence of nocturia and voiding symptoms in females <55 years old whereas no significant relationship was found in CCB use among males. On the contrary, one study [19] reported an inverse association of CCB use with LUTS.

### 3.4. Impact of Calcium Channel Blockers-Related Lower Urinary Tract Symptoms on the Quality of Life

As shown in Table 2, 2 out of 5 studies did not assess the impact of CCB-related LUTS on the QOL [17, 19]. Hughes et al. reported a significant increase in the mean QOL score after CCB commencement [15]. This finding indicated a significant reduction in the individuals' QOL after CCB therapy as the overall inconvenience to participants caused by their current urinary tract symptoms (IPSS-QOL index) was obtained by scoring from 0 (delighted) to 6 (terrible). Moreover, Elhebir also revealed that non-CCB-users had better QOL than CCB-users [16]. By contrast, one study reported that there was no significant difference in the QOL among individuals on CCB therapy and untreated hypertensives [18].

### 3.5. Quality Assessment

The quality assessment of studies using NOS is shown in Table 3. The qualities of studies were considered high for four studies [16–19] and low for one study [15].

## 4. Discussion and Interpretation

This systematic review was sought to examine the association of CCB with LUTS in humans and the impact of this association on patients' QOL. Despite a high prevalence of hypertension (40% in adults aged ≥25 years [20]) worldwide and a significantly high use of CCB, data regarding the association of CCB use with LUTS and their impact on individuals QOL is sparse as we were able to identify only five studies that met the inclusion criteria. Among the included studies, three reported a significant relationship between CCB use and LUTS [15, 16, 18] whereas, in the remaining two studies, one study [17] reported that monotherapy of CCB was linked with higher prevalence of nocturia and voiding symptoms in only young females and the other study [19] reported the inverse association of CCB use with LUTS. Of three studies that reported the association of CCB with LUTS, one study [15] had a very small sample size (*N* = 38 males) to show any differences in CCB subclass [dihydropyridines (DHP) versus nondihydropyridines (NDHP)] effects or any differences in the effects of individual CCB within the subclasses, and the outcome depended on participants' recall of their urinary symptoms before the initiation of CCB therapy, which in some cases (39.5%) was greater than 5 years earlier. Moreover, 18 out of the 38 participants had medical conditions (stroke, spinal disc disorders, congestive heart failure, impaired mobility, recurrent cough, and transurethral resection of the prostate) that potentially contribute to LUTS. Another study that demonstrated a significant increase in the mean IPSS score in CCB-users as compared to untreated hypertensives did not describe the types of CCB (DHP or NDHP, monotherapy or CCB combination with other antihypertensive agents, and the individual CCB) [18]. Interestingly, this study reported that angiotensin receptor blockers may have the potential to improve LUTS in men. Only the study conducted by Elhebir [16] demonstrated the impact of NDHP (diltiazem and verapamil), highly vascular selective DHP (felodipine and lercanidipine), and other DHP (amlodipine and nifedipine) on LUTS. They reported that felodipine and lercanidipine were not associated with LUTS, whereas amlodipine, nifedipine, diltiazem, and verapamil were found to have significant association with severe as well as moderate-severe LUTS. Moreover, they also reported that a significantly higher number of CCB-users in their study were found to be taking medications for their urinary symptoms (22.4% versus 9.3%, *p* = 0.003) and have had urogenital surgeries compared to the non-CCB-users (16.5% versus 7.8%, *p* = 0.029). These findings demonstrated the extra burden on these patients as well as their families. The impact of the CCB induced LUTS on patients' QOL was controversial as the findings of Ito et al. [18] were contradicting to the findings of Hughes et al. [15] and Elhebir [16] reporting the significant worsening in the patients' QOL due to the precipitation and exacerbation of LUTS due to CCB.

## 5. Limitations

Though all possible efforts were made to warrant the inclusion of all potentially relevant research studies in this review, unintentional selection biasness might still be present. Furthermore, our literature search yielded 2023 publications and only five were deemed appropriate for the inclusion in the review, not enough for formal quantitative meta-analysis. The association of CCB with LUTS was not the primary objective in some of the selected studies and the majority of the included studies did not specify the types of CCB.

## 6. Recommendations

Due to the advancement in clinical research, many new CCB medications are now available in the market. These medications contain CCB combination with other drugs (diuretics, angiotensin enzyme inhibitors, angiotensin receptor blockers, statins, etc.). The findings of the current systematic review warrant further investigations using a large sample size to explore the effect of CCB in precipitation and exacerbation of LUTS (DHP versus NDHP, CCB monotherapy versus CCB combination therapy, and effects of individual CCB within the subclasses on LUTS).

## 7. Conclusion

The findings of the present review indicated that despite the extensive CCB use to treat various cardiovascular diseases worldwide, there is limited data concerning the association of these agents with urinary symptoms. However, further studies are required to provide concrete evidence about the said association and its impact on patients' QOL. Healthcare providers should make efforts for an early identification of the individuals at risk of LUTS prior to the commencement of CCB therapy. Moreover, patients should be counselled to notify their healthcare provider if they notice urinary symptoms after the initiation of CCB therapy.

## Figures and Tables

**Table 1:** Description of the five studies included in this systematic review of studies evaluating the relationship of calcium channel blockers with lower urinary tract symptoms.

Author [ref] year	Country	Study design	Study population	Number of participants, age, gender	Study objective	Research instrument
Hughes et al. [15] 2011	Australia	Retrospective cohort study	Participants from community pharmacies and a medical practice in the southwest suburbs of Perth, Western Australia	*N* = 38, age ≥ 45 yrs (mean age: 66.9), 100% males	To determine the effect of calcium channel blockers on lower urinary tract symptoms	International Prostate Symptom score, American Urological Association Benign Prostatic Hyperplasia Impact Index, Symptom diary

Elhebir [16] 2011	Australia	Cross-sectional study	Participants admitted to Royal Perth Hospital general medicine wards	*N* = 278, age ≥ 40 yrs (mean age: 72.1), 54.3% males	To evaluate the relationship between calcium channel blockers use and lower urinary tract symptoms in general medical inpatients	International Prostate Symptom score

Hall et al. [17] 2012	USA	Cross-sectional study	Participants from The Boston Area Community Health Survey	*N* = 1865, age 30–79 yrs (mean age: 55.2), 54% males	To examine differences in the prevalence of lower urinary tract symptom among users of five common antihypertensive medication classes compared with nonusers	American Urological Association—Symptom Index

Ito et al. [18] 2013	Japan	NR	Participants from a multicenter Japanese study on silodosin	*N* = 3790, NR (mean age: 71.1), 100% males	The associations between male lower urinary tract symptoms and hypertension, and to examine whether antihypertensive medications, particularly Angiotensin-II receptor blockers, influence LUTS	International Prostate Symptom score

Kok et al. [19] 2009	Netherlands	Longitudinal population-based study	Participants from longitudinal, community based Krimpen Study of male urogenital tract problems and general health status	*N* = 1668, age 50–78 years old (mean age: 60.6), 100% males	To explore the risk factors for lower urinary tract symptoms suggestive of benign prostatic hyperplasia in a community based population of healthy aging male	International Prostate Symptom score

NR: not reported.

**Table 2:** Association of calcium channel blockers with lower urinary tract symptoms.

Author [ref] year	Number of CCB-users	Types of CCB evaluated	Association of CCB with LUTS	Quality of Life
Hughes et al. [15] 2011	38	Amlodipine, felodipine, nifedipine, lercanidipine, diltiazem, and verapamil	After adjusting for the natural progression of LUTS, there was a significant increase in mean IPSS after CCB initiation (5.85; 95% CI: 4.26–7.45, *p* < 0.001)	Significant increase in mean IPSS-QOL score after CCB commencement (2.27; 95% CI: 1.40–3.15, *p* < 0.001).

Elhebir [16] 2011	85	Amlodipine, felodipine, nifedipine, lercanidipine, diltiazem, verapamil, Amlodipine/diltiazem, and felodipine/verapamil	CCB-users more likely to suffer from moderate-severe LUTS than non-CCB-users (*p* < 0.001) High significant association of Amlodipine/nifedipine (9.8; 95% CI: 3.98–24.3, *p* < 0.001) and diltiazem/verapamil (8.2; 95% CI: 1.93–34.9, *p* = 0.004) with severe LUTS Significantly higher odds of moderate-severe LUTS with CCB (amlodipine/nifedipine, and diltiazem/verapamil [37.5; 95% CI: 8.6–163.9, *p* < 0.001])	CCB-users had statistically significantly higher scores of Benign prostate hyperplasia impact index (*p* = 0.017) and IPSS-QOL question (*p* < 0.0001).

Hall et al. [17] 2012	Monotherapy 54, CCB + other AHT 153	NR	Monotherapy of CCB is associated with higher prevalence of nocturia (OR 2.65; 95% CI: 1.04–6.74, *p* = 0.03) and voiding symptoms (OR 2.59; 95% CI: 1.24–11.87, *p* = 0.05) in young females (<55 years); no associations of CCB (monotherapy and CCB use with other AHT) with LUTS in males	NR.

Ito et al. [18] 2013	206	NR	Mean IPSS score was significantly high in CCB-users than nontreated hypertensives (19.6 versus 18.2, *p* < 0.05)	No difference of QOL scores between CCB-users and nontreated hypertensives.

Kok et al. [19] 2009	39	NR	Preventive effect of CCB use in development of LUTS suggestive of BPH (HR 0.38; 95% CI: 0.151–0.979, *p* = 0.04)	NR.

AHT: antihypertensive; CCB: calcium channel blockers; LUTS: lower urinary tract symptoms; QOL: quality of life; HR: hazards ratio; OR: odds ratio; NR: not reported.

**Table 3:** Study quality assessment using Newcastle-Ottawa scale.

Study [ref] year	Selection	Comparability	Outcome	Total score
Hughes et al. [15] 2011	*∗*	*∗∗*	*∗*	4
Elhebir [16] 2011	*∗∗∗∗∗*	*∗∗*	*∗∗*	9
Hall et al. [17] 2012	*∗∗∗∗*	*∗∗*	*∗∗*	8
Ito et al. [18] 2013	*∗∗∗∗*	*∗∗*	*∗∗*	8
Kok et al. [19] 2009	*∗∗∗∗∗*	*∗∗*	*∗∗*	9
